# Socioeconomic Factors in Brain Research: Increasing Sample Representativeness with Portable MRI

**DOI:** 10.1017/jme.2024.168

**Published:** 2024

**Authors:** Martha J. Farah

**Affiliations:** 1: UNIVERSITY OF PENNSYLVANIA, PHILADELPHIA, PA, USA

**Keywords:** Health Disparities, Inequality, Poverty, Research Participation, Cognition, Community-Based Research

## Abstract

People of low socioeconomic status (SES) are often underrepresented in biomedical research. The importance of demographically diverse research samples is widely recognized, especially given socioeconomic disparities in health, but have been challenging to achieve. One barrier to research participation by low SES individuals is their distance from research centers and the difficulty of traveling. This article examines the promise of portable magnetic resonance imaging (pMRI) for enrolling participants of diverse SES in structural neuroimaging studies, and anticipates some of the challenges, practical and ethical, that may arise in the course of such research.

## Introduction

Until recently, participants in magnetic resonance imaging (MRI) research were obliged to travel to the site of an MRI scanner. The enormous weight and exacting infrastructure requirements of these scanners has generally required that they be installed in a fixed location. Portable MRI (pMRI) scanners overcome this requirement, albeit with differences from fixed scanners. Shen et al. (2024) review some of these differences.^1^ First, the main magnetic field in pMRI systems is weaker than in fixed scanners, and image quality is accordingly worse. However, new artificial intelligence (AI)-driven analytic approaches can recover additional information from pMRI scans, increasing the utility of the scans. Second, the technical capabilities of pMRI are likely to develop further. Third and most relevant, pMRI opens new possibilities for research with participants who cannot easily travel to a fixed scanner.

We can therefore anticipate that pMRI will increase the diversity of research samples, including elderly participants with cognitive impairment for whom travel to a university or medical center may be challenging^2^ and participants at a geographic remove from MRI centers or who lack the time or transportation options to make participation at a centralized research facility feasible. The latter population includes people of low socioeconomic status (SES). The next two sections include a brief overview of SES and its relevance to basic neuroscience research and translational biomedical research. Following this, some of the practical and ethical problems that arise when recruiting low SES individuals as research participants in MRI research are discussed.

## I. What is SES and How Is It Relevant to Neuroimaging Research?

Socioeconomic status refers to a bundle of moderately correlated factors,^3^ typically measured by degree of financial comfort versus need and access to various social resources such as education, working conditions, occupational prestige and neighborhood quality.^4^ Although participant age and gender are invariably reported in published descriptions of neuroscience research samples, SES is less consistently reported and rarely analyzed as a moderator of results.^5^ When researchers do report SES, they generally rely on a brief assessment with one or two measures, most commonly income and educational attainment,^6^ a defensible if not ideal strategy given Rakesh, Zelesky & Whittle’s (2021) finding that different SES measures yielded, in their words, “similar but distinct” patterns of resting functional MRI (fMRI) connectivity.^7^

SES is correlated with almost every type of illness, including cardiovascular health, metabolic health, immune function, cancer initiation and progression, dementia and mental illness.

Of course, the mere measurement of SES in research samples is not enough; an appropriate distribution of SES is essential for the external validity of the research.^8^ No matter how thorough the assessment of SES, validity will be compromised if the sample deviates from true population characteristics. In the words of Hyde et al. (2015), “[m]uch of the existing corpus of human neuroimaging research uses convenience and snowball sampling, often on college student populations. Thus, much of what we know about ‘the brain’ is actually based on collegiate and middle-class American and Western European brains, who differ in many concrete ways from others within America and those in the rest of the world.”^9^

How greatly do samples of convenience differ from the demographics of the US? Consider these recent statistics on income and education in the US: 11% of households in the US were below the poverty line in 2022, exemplified as less than $15,000 per year for single persons and less than $30,000 for families of four.^10^ For educational attainment in the same year, 9% of people aged 25 years or older had not graduated high school or earned a General Education Diploma (GED). Samples used in basic neuroimaging research rarely include participation by these segments of the population.

More representative sampling has been a goal of recent projects that provide large numbers of brain images and other participant data as a resource for research on a wide range of topics. These include the Pediatric Imaging Neurocognition and Genetics project (PING)^11^ and the Human Connectome Project,^12^ both of which include over a thousand participants, and the Adolescent Brain and Cognitive Development project (ABCD),^13^ and UK Biobank,^14^ which include images from over 10,000 and almost 70,000 respectively. The data sets just mentioned are all described as somewhat skewed toward higher-income participants with more education, despite concerted efforts to target recruitment at lower SES.^15^

The effect of sample composition on research findings can be seen in the results of an analysis of the PING data set by LeWinn et al. (2017),^16^ to be described in some detail here as a demonstration of how radically sample demographic distribution can affect results. LeWinn and colleagues assessed developmental change in brain structure between the ages of 3 and 18 years in the healthy children of the PING study. Although the sample included a wide range of SES as well as diversity of gender, race and ethnicity, the distribution of participants within those ranges nevertheless differed from the national distribution. PING participants were more likely to be Hispanic, come from higher-income homes and be cared for by more-educated caregivers than a national sample. LeWinn and colleagues performed two sets of analyses on the data, one with original data and one with population weighting to partially correct for deviations from population demographics.

The two analyses yielded different results, which is perhaps not surprising given that they were performed on slightly different data sets. However, some of the differences were both surprising and meaningful. Beyond finding differences in overall rate of brain growth and age of peak brain size, additional differences emerged, with implications for our understanding of normative brain development. The original, unweighted PING sample showed surface area in the parietal cortex reaching maximum size first, followed by the remaining three lobes (frontal, occipital and temporal), following very similar developmental courses to one another. This is inconsistent with the widely recognized later developmental course of frontal cortex, specifically, and the back-to-front sequence expected on the basis of other research. In contrast, the weighted sample produced results more consistent with expectations, showing occipital and parietal cortex expanding in surface area first, followed by temporal cortex and finally by frontal cortex.

Another generalization about brain development, which applies across species, is that subcortical structures mature before cortical structures. This pattern was not observed in the original sample but was found with the weighted sample. Finally, differences were found in the shape of the trajectory of brain development across ages. Although both samples showed curvilinear trends in their pattern of growth, these were quadratic for the original sample (that is, increased and then decreased like an upside-down U) and cubic for the weighted sample (that is, increased, then decreased, and finally began to reverse again, like a sideways S). These developmental trajectories have been interpreted with respect to the cellular processes that may underlie brain development, for example reductions interpreted as use-dependent synaptic pruning^17^ and to indicate possible windows of opportunity for protecting or intervening on brain development.

What are the implications of LeWinn et al.’s (2017) findings for basic research on the normal, healthy brain? They show that sample composition affects research conclusions. Underrepresentation of low SES participants affects not just single global parameters of the results (e.g., the rates of brain growth or asymptotic values in LeWinn’s study), but more detailed and multivariate relations among these parameters as well (e.g., relations among rates of growth for different brain regions, curvilinear patterns of growth). This presumably occurs by way of heterogeneity in measures of interest and their correlates across well-sampled and under-sampled groups. As Si et al. (2023) have pointed out, population weighting is an imperfect solution to this problem,^18^ and Gard et al. (2023) have discussed the many consequential effects of different sampling methods in basic science investigations and in applied health research.^19^

## II. SES and Brain Imaging for Health Research

In health research, where the goals include improved understanding, diagnosis and treatment of illness, socioeconomically diverse samples may be especially important. SES is correlated with almost every type of illness, including cardiovascular health, metabolic health, immune function, cancer initiation and progression, dementia, and mental illness.^20^

SES not only predicts disease prevalence, but in some cases predicts disease subtypes and treatment response. For example, in the realm of physical illness, SES correlates with subtypes of breast cancer independent of race,^21^ with implications for treatment. In the realm of mental illness, depression risk is correlated with SES, and SES-related depression may be distinctive in terms of anatomical correlates and the role of stress.^22^ The effectiveness of particular treatments for depression may also depend on SES.^23^

Why would brain imaging be relevant to SES gradients in such a wide range of diseases affecting organ systems other than the brain? The answer lies in the role of stress and its downstream physiological effects, coupled with the generally higher levels of stress that accompany lower SES. The brain plays a central role in stress physiology, most notably the hippocampus and prefrontal cortex.^24^ Psychosocial stress (as opposed to physical stress such as starvation or cold exposure) is transduced by the brain and has a well-documented impact on many facets of the immune system. These immune system changes result in greater susceptibility to infection,^25^ cardiovascular disease,^26^ Type 2 diabetes,^27^ cancer initiation and progression,^28^ and mood disorders.^29^

Unfortunately, individuals of low SES are typically underrepresented in biomedical research.^30^ Why has it been challenging for biomedical researchers to obtain socioeconomically inclusive samples? Multiple factors are undoubtedly at work, and many of them reflect differences in life circumstances for people of higher and lower SES. These differences include where people live (residential segregation), whom they know (minimally overlapping networks of friendship and acquaintance) and the institutions within which they spend time (both occupational and educational). These separations have become starker over recent decades in the U.S.^31^ and they present challenges for the recruitment of lower SES research participants.

Geographical separation is a major impediment to research participation for those from lower SES communities. Davis et al. (2019) found that transportation to university or medical center research sites is a major barrier to participation for those from low-income communities. They quote a medical resident as saying “[i]t’s hard enough for our patients to keep medical appointments without having to come back to participate in a clinical trial.”^32^ For this problem, at least, pMRI offers a promising solution: locating research in areas proximal to participants’ homes, schools and workplaces. However, once the challenge of geographic separation has been addressed, other challenges will become apparent. Some of these are reviewed in the next section.

## III. Challenges Beyond Geographic Separation

Portable MRI helps surmount a major practical barrier for research with low SES individuals by bringing the scanner to the research participants. However, many other challenges remain. These include problems to be addressed in pMRI research with any population, and problems to be addressed with any research involving low SES participants. Table 3 of Shen et al. (2024) enumerates 15 challenges that must be met for any research using pMRI. Several of them are particularly important for research in low-SES communities, with numbers 5, 6, 9, and 14 most directly relevant.^33^ Emery, Silverman & Carey (2023) review problems that arise in research of any kind with low SES participants.^34^ Because researchers rarely come from low SES communities themselves,^35^ some of these problems may not be foreseen.

Among the practical challenges beyond transportation are those directly related to financial factors. For example, home addresses may change more frequently for low SES individuals because of evictions and temporary stays in shelters or with family members. Mobile phone numbers may be deactivated for nonpayment or changed to avoid collection calls. These factors will make follow-up reminders less effective, and longitudinal studies will be more difficult. Drop-out would be expected in these circumstances, and it would likely be nonrandom with respect to participants’ socioeconomic characteristics.

The poor also have less control over their time. Work hours in low-wage jobs are less flexible and less predictable, on average, than in better paying jobs. Unpredictable events, from housing disruptions to lack of bus fare when needed for an appointment, will complicate scheduling appointments and keeping them.

Participant payment is another aspect of research that may be more complex with low-SES participants. The likelihood of being “unbanked” rises as SES drops, which adds complications to the mechanics of compensation.^36^ Additionally, participants may worry that reporting of payments by the research institution could lead to taxes owed or loss of eligibility for government assistance programs.

An important nonfinancial factor standing in the way of participation by lower SES individuals is trust. Trust of individuals and institutions is generally lower within lower SES communities.^37^ Compounding this, if a prospective participant has no experience with research projects and is unfamiliar with the idea of research as distinct from healthcare, apprehension about research participation would be understandable. Furthermore, while participants of any social class may fail to understand risk and safety information included in the consent form, those of low SES will be less likely to ask questions of the researcher.^38^ As a result, they may decline to participate, or worse, participate under perceived duress in a state of anxiety.

Shen et al. (2024) note that the problems just reviewed can be ameliorated by community engagement.^39^ Community engagement, as opposed to traditional “outreach” for health and science communication, involves bidirectional communication. By developing collaborative relationships with community members, researchers gain insight into feasible and socially acceptable ways to sustain contact, provide payment for participation and facilitate scheduling. They will also learn about possible sources of mistrust and adapt their communication and research to address issues of concern more clearly. In addition, the very existence of community collaborators will encourage trust.

Two other issues in human subject ethics are of particular relevance to participants of low SES. First, determining whether a participant has given informed consent may be more difficult when a power asymmetry exists between the researcher and participant. Some guidelines on consent classify as “vulnerable” all economically or educationally disadvantaged participants, postulating the need for additional protections in the consent process (although the nature of the assumed vulnerability and whether it can be applied categorically to all members of these categories is not clear).^40^

Second, participant payment becomes ethically more fraught when participants’ need for money is more intense and hence more motivating. We assume that there is a line between fair compensation for a participant’s time and effort and remuneration so extravagant that it would be hard to turn down by declining to participate. Where that line is will depend on the participant’s situation and may best be determined in consultation with the community.

A different set of issues arise in connection with research on SES itself, rather than other biomedical topics studied with socioeconomically inclusive samples. The goal of neuroscience studies of SES is to understand the causes and consequences of SES differences.^41^ Portable MRI would greatly facilitate sample acquisition for this growing research field.^42^ However, such research raises additional ethical issues, not within the scope of “research ethics” as normally understood.^43^ Instead, they relate to the potential for misinterpretation or misuse of the research findings to support the pathology or biological inferiority of certain groups.

The poor have long been stereotyped as less intelligent, less disciplined and less deserving of material and social success than their better-off counterparts.^44^ Differences in brain anatomy invite (false) conclusions about “built in” differences in intelligence and personality across levels of SES. The existence of a positive relation between brain size and SES could appear to supply additional evidence that negative stereotypes of low SES people are true. Furthermore, the common (but also false) belief that brain structure is unchangeable would invite the fatalistic belief that the traits mentioned above have a fixed relation to SES.

Of course, brain structure is not immutable, but is known to change in response to experience. Experience-driven plasticity is highest in early life but continues throughout all life stages.^45^ More relevant to the present issue, there is reason to believe that experience is in part responsible for SES differences in brain structure.^46^ Therefore, the existence of structural brain correlates of SES in no way implies immutable differences, or differences destined to be inherited by later generations.^47^

Whether to improve sample representativeness or to deepen the understanding of SES itself, pMRI offers unprecedented access to the imaging of socioeconomically diverse populations. Especially when SES is the focus of the research, authors must guide readers on the scientific interpretation of imaging results. This is preferable to abandoning or suppressing such research out of concern for biased misinterpretation.^48^

## Conclusion

The focus of this article has been on the many challenges, practical and ethical, that must be confronted once pMRI is deployed to study SES and health more generally. By identifying problems, with the goal of solving them, let us not conclude that pMRI is no more than a source of problems. As we acknowledge the need to modify some of our research practices, embrace collaboration with community members and communicate sensitively, we should not lose sight of what a huge advance pMRI is in population neuroscience research.
